# Antibodies to SARS-CoV-2 protect against re-infection during outbreaks in care homes, September and October 2020

**DOI:** 10.2807/1560-7917.ES.2021.26.5.2100092

**Published:** 2021-02-04

**Authors:** Anna Jeffery-Smith, Nalini Iyanger, Sarah V Williams, J Yimmy Chow, Felicity Aiano, Katja Hoschler, Angie Lackenby, Joanna Ellis, Steven Platt, Shahjahan Miah, Kevin Brown, Gayatri Amirthalingam, Monika Patel, Mary E Ramsay, Robin Gopal, Andre Charlett, Shamez N Ladhani, Maria Zambon

**Affiliations:** 1 National Infection Service Public Health England, London, United Kingdom; 2 London Coronavirus Response Centre, Public Health England, London, United Kingdom; 3 Data and Analytical Sciences, Public Health England, London, United Kingdom

**Keywords:** COVID-19 outbreak, care homes

## Abstract

Two London care homes experienced a second COVID-19 outbreak, with 29/209 (13.9%) SARS-CoV-2 RT-PCR-positive cases (16/103 residents, 13/106 staff). In those with prior SARS-CoV-2 exposure, 1/88 (1.1%) individuals (antibody positive: 87; RT-PCR-positive: 1) became PCR-positive compared with 22/73 (30.1%) with confirmed seronegative status. After four months protection offered by prior infection against re-infection was 96.2% (95% confidence interval (CI): 72.7–99.5%) using risk ratios from comparison of proportions and 96.1% (95% CI: 78.8–99.3%) using a penalised logistic regression model.

In autumn 2020, two care homes in London, United Kingdom (UK) with high rates of severe acute respiratory syndrome coronavirus 2 (SARS-CoV-2) seropositivity following outbreaks in the first wave of the coronavirus disease (COVID-19) pandemic [1,2] experienced a second COVID-19 outbreak. Outbreak investigations and SARS-CoV-2 serology were repeated to assess the role of antibodies in protecting against SARS-CoV-2 re-infection.

## Affected care homes

Care home A provides residential and dementia care for a maximum of 52 residents (median age 84 years; interquartile range (IQR): 76–89; 33/46 female at the time of the second outbreak). Serological investigations in June 2020 found 33/66 (50.0%) had SARS-CoV-2 antibodies after the first outbreak (18/32 residents; 15/34 staff).

Care home L provides residential and nursing care for a maximum of 64 residents (median age 85 years; IQR: 78–89; 36/57 female at the time of the second outbreak). Serological investigation in May 2020 identified 59/117 (50.4%) as seropositive (26/52 residents; 33/65 staff).

## Laboratory investigations

Nasal swabs were subjected to SARS-CoV-2 RT-PCR at the Public Health England (PHE) national reference laboratory as described previously [3]. Serological testing was conducted using in-house native virus lysate (PHE, UK) and receptor binding domain (RBD) EIA assays (PHE, UK), and a commercial nucleocapsid (N) assay (Abbott, Illinois, United States) [1,2,4]. Seropositivity was determined by reactivity in any assay; > 80% of samples were positive in ≥ 2 assays. The native virus lysate assay was the most sensitive assay [4]. Neutralising antibody titres were determined by live virus neutralisation [2].

Whole genome sequencing was attempted on all RT-PCR-positive samples tested at the PHE reference laboratory as described previously [3]. Completed viral genomes were deposited in GISAID (Supplementary Table).

Protective effectiveness was estimated using two methods: risk ratios (RR) from a comparison of proportions (Fisher’s exact test), and odd ratios (OR) from a penalised logistic regression model (Wald test).

A COVID-19 case was defined as any individual testing positive by RT-PCR for SARS-CoV-2, whether tested as a result of symptoms or through routine care home screening [5]. A re-infection was defined as an individual testing SARS-CoV-2 RT-PCR positive while having evidence of previous seropositivity by any assay, or a previous RT-PCR-positive result more than 90 days earlier in an individual without serological analysis (assumed to have seroconverted).

### Ethical statement

PHE has legal permission, provided by Regulation 3 of the Health Service (Control of Patient Information) Regulation 2002, to process patient confidential information for national surveillance of communicable diseases. The Investigation Protocol was reviewed and approved by the PHE Research Ethics and Governance Group (REGG) (Reference NR0204). Verbal consent for testing was obtained by care home managers from staff members and residents or their next of kin as appropriate.

## Outbreak evolution

**Figure 1:**
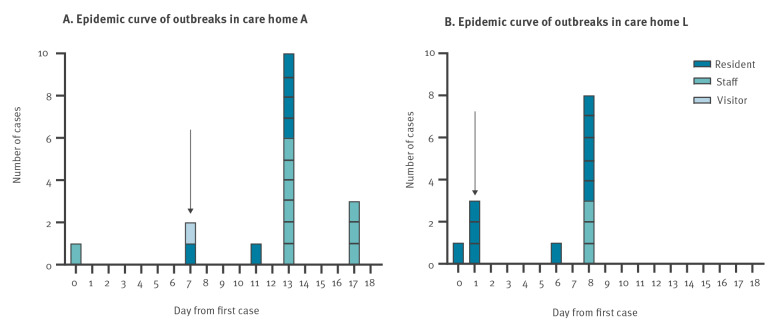
Epidemic curves of outbreaks in care home A (A) and care home L (B), London, United Kingdom, September and October 2020

The outbreak in care home A began with a symptomatic staff member in mid-September 2020 (Figure 1A). Subsequent COVID-19 cases were identified in an asymptomatic visitor and asymptomatic resident on routine whole home screening 7 days later, prompting the declaration of an outbreak and instigating day 0 and 7 mass testing as per national recommendations, with clearance testing at day 28 prior to returning to routine screening patterns [5]. One further resident was identified following a swab taken for ‘non-specific decline’. All other RT-PCR-positive individuals were identified through the mass outbreak screening conducted as feasible, depending on staff shifts, and all of them were asymptomatic throughout. Of 83 individuals (46 residents, 37 staff) that were swabbed,16 (6 residents, 10 staff) were RT-PCR positive, of whom two residents died, both within 1 week of testing positive. All but one of the COVID-19 cases were either seronegative (n = 7) or had unknown antibody status (n = 8) at the time of RT-PCR testing during the outbreak. The single previously seropositive staff member who was RT-PCR-positive is described below.

Mass testing occurred on day 0 and 7 as per national recommendations [5]. Clearance testing was conducted on day 28, no new RT-PCR confirmed cases were identified in either care home on day 28. These homes then returned to routine testing. Arrow indicates day outbreak declared.

The outbreak in care home L began in October 2020. The index case was a resident identified on screening following hospital admission for a non-COVID-19 related condition and lethargy (Figure 1B). Following the identification, within a few days three asymptomatic residents were subsequently identified through routine whole home testing. Two of the residents went on to develop symptoms and the third became unresponsive and was admitted to hospital where they subsequently died 1 week after testing positive. One further resident was identified as positive following a swab taken for lethargy and weakness. All other RT-PCR-positive individuals were identified through whole care home outbreak screening. All staff identified as positive remained asymptomatic throughout. Residents identified as RT-PCR-positive exhibited a range of non-specific symptoms including weakness, lethargy and loss of appetite. In total, 126 individuals (57 residents, 69 staff) were swabbed during the course of the mass outbreak screening; 13 (10 residents, 3 staff) were RT-PCR positive. All RT-PCR-positive residents and staff were either seronegative by all assays (n = 11) or had unknown antibody status (n = 2) at the time of RT-PCR testing.

There were no new COVID-19 cases identified in either care home on day 28. These homes then returned to routine testing. The outbreaks were declared over at the end of October and beginning of November 2020 in homes A and L, respectively.

## Genomics analysis

**Figure 2:**
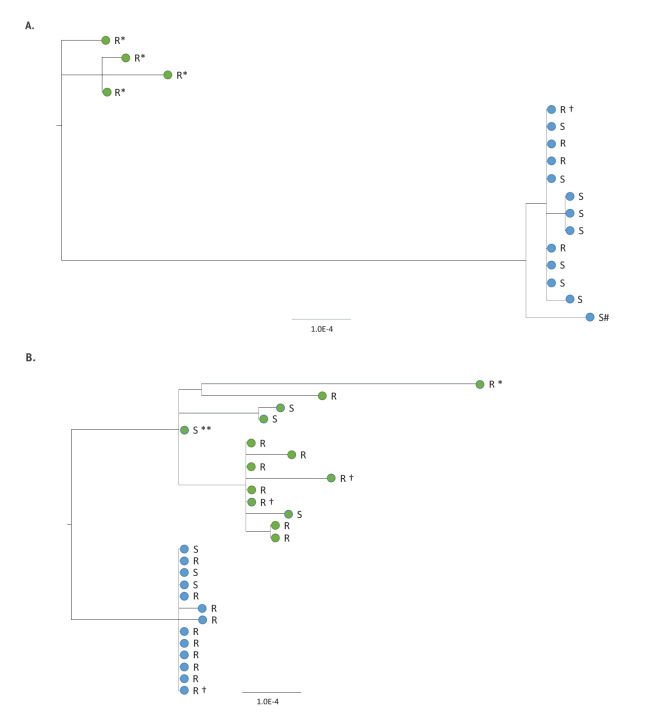
Maximum likelihood phylogeny of SARS-CoV-2 genomes from individuals in care home A (panel A) and L (panel B) involved in the second outbreak in comparison to genomes from the first outbreak

The second COVID-19 outbreaks experienced by both care homes were due to SARS-CoV-2 strains that were genetically distinct from their respective first outbreaks which occurred during the first wave of the pandemic in spring 2020 (Figure 2). In care home A, virus strains from the earlier outbreak had S gene 614D, whereas the strains in the later outbreak were 24–27 single nucleotide polymorphisms (SNPs) different and contained S gene 614G. In the second outbreak, nine individuals were infected by an identical strain, which differed by 1–2 SNPs from three other COVID-19 cases. The individual with a probable re-infection (S#) shared a virus sequence from B1.36 lineage and the same UK1350_1.2.1.1 phylotype as the other residents and staff, with 6 SNPs differences from the main cluster, including three mixed bases which were all outside the S protein RBD coding region (Figure 2A). In care home L, virus strains from the earlier outbreak arose from several introductions and contained a mixture of 614D and 614G strains, whereas the second outbreak strains were all S gene 614G and differed by 11–18 SNPs from earlier strains (Figure 2B). In both care homes, fatal cases in residents had identical viral genomes to surviving residents.

R: Resident; S: Staff member; Ϯ: Fatality; *: S gene 614D variant; **: S gene 614D/G mixed base variant.

Unless otherwise specified all virus are 614G variant SARS-CoV-2 viruses.

Panel A: care home A, first outbreak in late April / early May 2020 (green dots) and second outbreak in late September / early October 2020 (blue dots).

Panel B: care home L, first outbreak in April 2020 (green dots) and second outbreak in October 2020 (blue dots).

Viral amplicons were sequenced using Illumina library preparation kits (Nextera kit, Illumina, Cambridge, UK) and sequenced on Illumina short-read sequencing machines (Nextseq or Hiseq sequencing platforms, Illumina, Cambridge, UK). The bioinformatics protocol to generate consensus sequences utilised Trimmomatic, BWA (mapping), and an in-house variant caller (quasibam) to align against a SARS-CoV-2 reference genome (NC_045512.2). Consensus sequences were generated using a depth cut-off of 20 reads and ambiguities called where a minority variant detected at ≥20%, these were aligned using MAFFT (Multiple Alignment using Fast Fourier Transform, version 7.310), manually curated and maximum likelihood phylogenetic trees derived using IQtree (version 2.04). Genomes were included in analysis where the coverage of the reference genomes was ≥80%. Completed viral genomes were deposited in GISAID (Supplementary Table).

## Serological analysis

Based on serological investigations conducted after the first outbreaks in these care homes in April and May 2020, there were 73 seronegative individuals (40 residents, 33 staff) across the two care homes. Eighteen (13 residents, 5 staff) were RT-PCR positive during the second outbreaks, 16 (11 residents, 5 staff) of whom had blood taken after these outbreaks and all seroconverted in at least two assays. Additionally, four individuals (2 residents, 2 staff), who were both seronegative before the second outbreaks and SARS-CoV-2 RT-PCR negative during these outbreaks, subsequently seroconverted.

Only one seropositive staff member in care home A tested SARS-CoV-2 RT-PCR positive during the second outbreak investigation. This individual had been RT-PCR negative during the first outbreak in the spring and remained asymptomatic throughout. The staff member was subsequently SARS-CoV-2 antibody positive on all three serological assays in June 2020 but did not have detectable neutralising antibodies. Two weeks after the positive RT-PCR test at the second outbreak in the autumn, the individual had boosting of both N and S antibodies and developed neutralising antibodies to a titre of 1:1,057.

## Attack rate and protective effectiveness of previous exposure

Only 1.1% (1/88) of individuals with confirmed previous SARS-CoV-2 exposure (antibody positive (n = 87) or RT-PCR positive (n = 1)) became PCR-positive during the second outbreaks compared with 24.7% (18/73) of those with confirmed seronegative status before the second outbreaks (Table). Considering also the four previously seronegative individuals who tested RT-PCR negative during the second outbreak but had seroconverted after the outbreak, this gives a combined attack rate of 30.1% (22/73). The estimated RR was 0.038 (95% CI: 0.005–0.273; p < 0.0001) (Fisher’s exact test) and the protective effectiveness estimate using RRs from a comparison of proportions [100*(1-RR)] was 96.2% (95% CI: 72.7%–99.5%). The estimated OR using a penalised logistic regression model was 0.039 (95% CI: 0.01–0.21; p < 0.001) (Wald test), with an effectiveness estimate [100*(1-OR)] of 96.1% (95% CI: 78.8%–99.3%).

**Table:** Severe acute respiratory syndrome coronavirus 2 attack rate by susceptibility status in two care home COVID-19 outbreaks, London, United Kingdom, September and October 2020

Category	Residents (n = 103)			Staff (n = 106)			Overall	Attack rate	Attack rate percentage
	Status pre outbreak	PCR positive during outbreak	PCR negative during outbreak, seroconversion	Status pre outbreak	PCR positive during outbreak	PCR negative during outbreak, seroconversion			
**Not susceptible**	44^a^	0	NA	44	1	NA	88/209	1/88	1.1%
**Susceptible**	40	13	2	33	5	2	73/209	22/73	30.1%
**Unknown**	19	3	NA	29	7	NA	48/209	10/48	20.8%

COVID-19: coronavirus disease; NA: not applicable.

^a^ Susceptibility of one case in this subset was determined by RT-PCR positivity and assumption of seroconversion as no serological result was available.

Susceptibility has been determined on basis of serological results before outbreaks using native viral antigen lysate EIA [2,4] or RBD [1] or Abbott Total N IgG, or the detection of RT-PCR positive without a corresponding serology. Two of the fatal cases were included in the susceptible cohort, whereas the third case had unknown susceptibility status.

## Discussion and conclusions

Care homes have been disproportionately affected by the COVID-19 pandemic, with high rates of infection and deaths among the frail, elderly residents [6,7]. Nearly all survivors, however, develop high SARS-CoV-2 antibody levels, including neutralising antibodies, after infection [2,8-10], but whether prior SARS-CoV-2 infection protects against re-infection is not known. In spring 2020, we investigated 13 London care homes, including the two care homes described here, and established prospective surveillance in this cohort of over 1,500 residents and staff. We identified high rates of SARS-CoV-2 infection among residents and staff during the first pandemic wave in the UK, most of whom were asymptomatic at the time of testing [3]. Follow-up serological assessments found that almost all RT-PCR-positive and two-thirds RT-PCR-negative residents and staff had SARS-CoV-2 antibodies, irrespective of age, sex or symptom status [1,2].

In July 2020, a national testing programme was implemented in care homes in the UK, with weekly swabbing for staff and 4-weekly swabbing for residents [11]. There were very few sporadic SARS-CoV-2 infections in these homes during the summer months but the two homes described here had second outbreaks during the autumn.

High rates of SARS-CoV-2 infection were detected in susceptible staff and residents during the second outbreaks in these two homes. Three of 16 susceptible SARS-CoV-2 RT-PCR-positive residents died. We found evidence of silent transmission, shown by seroconversion in RT-PCR-negative individuals, emphasising the continuing threat from SARS-CoV-2 in elderly people and the difficulty of maintaining control in closed settings. Prior infection with SARS-CoV-2 as determined by antibody or RT-PCR positivity was highly protective at 4 months. Only one re-infection occurred in a seropositive staff member, whose antibodies were boosted following re-infection. The first SARS-CoV-2 infection was not confirmed in this re-infection case, who was asymptomatic during both infections. This individual had N and S antibodies but did not have detectable neutralising antibodies following the first infection (data not shown), in keeping with other findings suggesting that functional antibody is an important correlate of protective immunity [12-14]. Taking all of these findings together highlights the importance comprehensive vaccination to protect vulnerable populations during the current pandemic.

Prior antibody testing with detailed public health and genomics investigations in the two care homes that experienced second COVID-19 outbreaks allowed the assessment of protection against re-infection, including protection against viruses with differences at position S 614, considered to be important for virus transmissibility [15].

## Supplementary Data

517000 Supplementary Material
