# Supplementary material for: Antibodies to SARS-CoV-2 protect against re-infection during outbreaks in care homes, September and October 2020
Source: Euro Surveill. 2021 Feb 4;26(5):2100092. doi: 10.2807/1560-7917.ES.2021.26.5.2100092 (PMC7863231; doi:10.2807/1560-7917.ES.2021.26.5.2100092)
Supplement: Supplementary Material [file 21-00092_Jeffery-Smith_Supplementary_Table.pdf]

This supplementary material is hosted by *Eurosurveillance* as supporting information alongside the article' Antibodies to SARS-CoV-2 protect against reinfection during outbreaks in care homes, September and October 2020', on behalf of the authors, who remain responsible for the accuracy and appropriateness of the content. The same standards for ethics, copyright, attributions and permissions as for the article apply. Supplements are not edited by *Eurosurveillance* and the journal is not responsible for the maintenance of any links or email addresses provided therein.

Supplementary Table. Lineage allocation and GISAID Accession Number [a]

GISAID_accession	Lineage (Pangolin 27/11/2020)	Care Home	UK Phylotype
EPI_ISL_528408	B.2.1	A	UK107_1
EPI_ISL_528409	B.2.1	A	UK107_1.132.1
EPI_ISL_528410	B.2.1	A	UK107_1.132
EPI_ISL_527358	B.2.1	A	UK107_1.132
EPI_ISL_622833	B.1.36.1	A	UK1350_1.2.1.1
EPI_ISL_622834	B.1.36	A	UK1350_1.2.1.1
EPI_ISL_622835	B.1.36.1	A	UK1350_1.2.1.1
EPI_ISL_622836	B.1.36.1s	A	UK1350_1.2.1.1
EPI_ISL_622837	B.1.36.1	A	UK1350_1.2.1.1
EPI_ISL_622838	B.1.36.1	A	UK1350_1.2.1.1
EPI_ISL_622839	B.1.36.1	A	UK1350_1.2.1.1
EPI_ISL_622840	B.1.36	A	UK1350_1.2.1.1
EPI_ISL_622841	B.1.36.1	A	UK1350_1.2.1.1
EPI_ISL_622842	B.1.36	A	UK1350_1.2.1.1
EPI_ISL_609840	B.1.36	A	UK1350_1.2.1.1
EPI_ISL_609841	B.1.36	A	UK1350_1.2.1.1
EPI_ISL_609842	B.1.36.1	A	UK1350_1.2.1.1
EPI_ISL_609885	B.1.1	L	UK2631_1

EPI_ISL_609886	B.1.1	L	UK2631_1
EPI_ISL_609887	B.1.1	L	UK2631_1
EPI_ISL_609888	B.1.1	L	UK2631_1
EPI_ISL_613571	B.1.1	L	UK2631_1
EPI_ISL_613572	B.1.1	L	UK2631_1
EPI_ISL_613573	B.1.1	L	UK2631_1
EPI_ISL_613574	B.1.1	L	UK2631_1
EPI_ISL_613575	B.1.1	L	UK2631_1
EPI_ISL_609898	B.1.1	L	UK2631_1
EPI_ISL_609901	B.1.1	L	UK2631_1
EPI_ISL_613598	B.1.1	L	UK2631_1
EPI_ISL_528430	B	L	UK473_1
EPI_ISL_466479	B.1.1	L	UK2420_1.4
EPI_ISL_466480	B.1.1	L	UK2420_1.4
EPI_ISL_466481	B.1.1	L	UK2420_1.4
EPI_ISL_466482	B.1.1	L	UK282_1
EPI_ISL_466483	B	L	UK282_1.3.1.1
EPI_ISL_466485	B.1.1	L	UK2420_1.4
EPI_ISL_466486	B.2.1	L	UK282_1.3.1.1.47
EPI_ISL_466487	B.1.1	L	UK3476_1.14
EPI_ISL_466488	B.1.1	L	UK2420_1.4
EPI_ISL_466489	B.1.1	L	UK2420_1.4
EPI_ISL_466490	B.1.1	L	UK2420_1.4
EPI_ISL_466491	B.1.1	L	UK3476_1.14

[a] Green highlighted genome from reinfection case.
